# Systemic Inflammation and Oxidative Stress in Adults with Bronchiectasis: Association with Clinical and Functional Features

**DOI:** 10.6061/clinics/2021/e2474

**Published:** 2021-04-07

**Authors:** Anderson Alves de Camargo, Rejane Agnelo Silva de Castro, Rodolfo P. Vieira, Manoel Carneiro Oliveira-Júnior, Amanda Aparecida de Araujo, Kátia De Angelis, Samia Zahi Rached, Rodrigo Abensur Athanazio, Rafael Stelmach, Simone Dal Corso

**Affiliations:** I Programa de Pos-Graduacao em Ciencias da Reabilitacao, Universidade Nove de Julho (UNINOVE), Sao Paulo, SP, BR; II Departamento de Ciencias do Movimento, Universidade Federal de Sao Paulo (UNIFESP), Santos, SP, BR; III Programa de Pos-Graduacao em Bioengenharia, Universidade Brasil, Sao Paulo, SP, BR; IV Departamento de Fisiologia, Universidade Federal de Sao Paulo (UNIFESP), Sao Paulo, SP, BR; V Divisao de Pneumologia, Instituto do Coracao (InCor), Hospital das Clinicas HCFMUSP, Faculdade de Medicina, Universidade de Sao Paulo, Sao Paulo, SP, BR

**Keywords:** Bronchiectasis, Exercise Test, Inflammation Mediators, Oxidative Stress

## Abstract

**OBJECTIVES:**

To compare the inflammatory and oxidative stress (OS) states of adults with bronchiectasis with those of healthy controls and correlate inflammatory and OS levels with lung function and physical capacity.

**METHODS:**

This study used a cross-sectional design. Seventy-four adults with bronchiectasis (age: 49±15 years, forced expiratory volume in 1 second [FEV1]: 52.5±25.6%) and 42 healthy controls (age: 44±17 years, FEV1: 95.9±14.0%) performed cardiopulmonary exercise tests and incremental shuttle walking tests. Their physical activity in daily life, inflammatory cytokine, and antioxidant levels in plasma were measured.

**RESULTS:**

Compared to that of the controls, the levels of interleukin (IL)-6 (*p*<0.001), IL-10 (*p*<0.001), carbonylated proteins (*p*=0.001), and superoxide anions (*p*=0.046) were significantly increased in adults with bronchiectasis. Catalase activity was also reduced in this group (*p*<0.001). The inflammatory markers IL-1β, IL-6, and tumor necrosis factor-α correlated negatively with aerobic capacity (r=-0.408, r=-0.308, and r=-0.207, respectively). We observed similar correlations with OS markers (thiobarbituric acid and carbonyls; r=-0.290 and r=0.379, respectively), and these markers also significantly correlated with the aerobic capacity.

**CONCLUSIONS:**

Adults with bronchiectasis presented an increased systemic inflammatory response that correlated negatively with physical capacity.

## INTRODUCTION

Bronchiectasis is a chronic lung disease with multifactorial causes characterized by abnormal and irreversible distortion of the bronchi. The structural changes in the bronchi include epithelial lesions that facilitate the retention of lung secretions. Consequently, there is an increase in bacterial colonization, chronic airway inflammation, and remodeling (1). This process leads to exacerbation of the disease, negatively affecting its prognosis and severity (1,2).

Chronic inflammation plays a central role in bronchial tree injury, inducing an exacerbated neutrophilic response (3) characterized by increased release of proinflammatory cytokines such as IL-6, IL-8 and TNF-α (4). Previous studies have demonstrated that increased cytokine levels in both plasma and bronchoalveolar lavage fluid are typical in adults with bronchiectasis (4- 7), even during the stable phase of the disease. Cytokine levels correlate with disease extension (5,6), lung function (8) and quality of life (5).

Oxidative stress (OS) also plays an important role in the pathogenesis of other chronic inflammatory pulmonary diseases such as asthma, chronic obstructive pulmonary disease, and cystic fibrosis. Although a complex interaction between OS and inflammation has been well established in several diseases, the role of OS in bronchiectasis has not been thoroughly investigated (9). In summary, OS drives signaling that activates inflammatory cells to release massive amounts of proinflammatory cytokines, perpetuating the inflammatory process (10). A few studies have demonstrated that individuals with bronchiectasis exhibit increased levels of some reactive oxygen species (ROS) in their plasma and exhaled breath condensate (11,12). Thus, increased rates of OS may be related to a typical chronic inflammatory syndrome in adults with bronchiectasis (12). On the other hand, increased levels of some antioxidants may occur as a response to elevated OS (12).

The inflammatory response is based on interactions that occur within a complex cytokine network and can be regulated at the pro- and anti-inflammatory levels (3). As observed in other chronic lung diseases, the impairment in functional capacity and the activities of daily living observed in individuals with bronchiectasis (13,14) can contribute to decreases in muscle performance, such as impairment of muscle regeneration, muscle atrophy, and lower muscular endurance (15). Therefore, it is reasonable to infer that the higher the inflammatory level or OS of patients with bronchiectasis, the lower their capacity for exercise and physical activity.

Previous studies have shown that worsening of the severity of the disease is related to high systemic levels of inflammatory mediators (6) and greater impairment of pulmonary function (4), quality of life (16), and exercise capacity (13). These findings demonstrate that individuals with severe disease have a worse prognosis. However, in our study, when we compared the inflammatory mediator and OS levels with the degree of disease severity, no significant associations were found, possibly due to heterogeneity in the etiology of the disease, differences in the airway microbiota (17), and the limited number of severely affected individuals studied.

Although comparisons between levels of inflammatory mediators (4-7) and OS (9,11-12) are found in the literature, a possible association among inflammatory cytokines, OS, and exercise capacity in adults with bronchiectasis has not yet been investigated.

In this study, we aimed to compare the inflammatory status and OS levels of adults with bronchiectasis with those of their healthy peers. We also aimed to correlate the inflammatory status and OS with pulmonary function, exercise capacity, and peripheral muscle strength in adults with bronchiectasis.

## METHODS

This study was a cross-sectional secondary analysis of data obtained in a randomized controlled trial (NCT02208830). Participants with obstructive pulmonary disease (from January 2014 to December 2015) at the general hospital of the School of Medicine of University, São Paulo (HCFMUSP) were included, and evaluations were performed at the Cardiopulmonary Rehabilitation Center of the Universidade Nove de Julho (UNINOVE). This study was performed in accordance with the principles set forth in the Declaration of Helsinki. Written consent to participate in the study was obtained from each participant, and the Ethics Committees approved the study (UNINOVE-451538 and HCFMUSP-0921/11).

Adults ≥18 years of age with a diagnosis of non-cystic fibrosis bronchiectasis confirmed by HRCT and the absence of pulmonary exacerbation for at least 4 weeks (defined by the presence of at least three of the following symptoms: change in quantity or consistency and color of secretion, increase in dyspnea symptoms, cough frequency or intolerance to exercise, and hemoptysis within 48 hours requiring antibiotic therapy), were enrolled in the study (18). Individuals presenting with current cardiopulmonary, musculoskeletal, or cognitive disease, those with a history of smoking (≥10 packs/year), and practitioners of physical activity (>2×/week) were excluded. The control group was recruited from the community and matched to the bronchiectasis group in terms of age, sex, and body mass index; the individuals in this group presented with normal lung function and no cardiopulmonary disease.

### Outcomes

Spirometric tests were performed using Ultima CPX (MedGraphics Corporation^®^, St. Paul, MN, USA) according to the recommendations of the Brazilian Consensus and the parameters registered and analyzed for the Brazilian population (19).

Functional capacity was measured using the incremental shuttle walking test (ISWT). The ISWT was performed twice on the same day (30 minutes apart), as described in another study (20).

The Cardiopulmonary Exercise Test (CPET) was conducted on an electromagnetically braked cycle ergometer with gas exchange and ventilatory variables analyzed breath-by-breath, as described in another study (21).

The number of steps taken by each individual was recorded using a pedometer device (Yamax Power Walker, model PW-610, Yamax Corp, Tokyo, Japan) in accordance with the manufacturer’s recommendations for 5 consecutive days. The data obtained on the first and last day were discarded, and an average of the data obtained on three consecutive days was considered in the analysis. The participants were instructed to carry the device in the right pocket on the anterior surface of their pants. They were instructed to remove the device only during bathing and at bedtime and maintain their usual activity levels on the days they wore the device. The individuals were classified as follows: inactive, <5000 steps/day; low activity, 5000-7499 steps/day; active, ≥7500 steps/day (22).

Dyspnea was evaluated using the modified Medical Research Council (mMRC) scale (23).

The severity of bronchiectasis was assessed using E-FACED, and the results were interpreted as follows: mild (0-3 points), moderate (4-6 points) and severe (7-9 points) (24).

### Systemic inflammatory and OS analysis

Plasma levels of interleukin (IL)-1β, IL-6, IL-8, IL-10, and tumor necrosis factor (TNF)-α were determined using commercially available enzyme-linked immunosorbent assay kits (R&D Systems Inc., Minneapolis, MN, USA) in accordance with the manufacturer’s instructions. The measurements were performed in the Spectramax i3 multiplate reader (Molecular Devices, CA, USA).

Protein, oxidatively modified protein, catalase activity, thiobarbituric acid reactive substance (TBARS), superoxide anion, total antioxidant capacity (TAC), hydrogen peroxide (H_2_O_2_), and nitrite concentrations were determined as described previously (APPENDIX).

### Statistical analysis

The data were analyzed using SPSS version 20.0 (SPSS Inc, Chicago, IL, USA). The Shapiro-Wilk test was used to evaluate the data distribution. Parametric and nonparametric data was presented as mean±standard deviation and as median and interquartile range, respectively. Differences between the groups were analyzed using the Mann-Whitney test. Correlations were analyzed using Spearman’s correlation. *p*<0.05 was considered significant.

## RESULTS

A total of 122 participants were evaluated. We excluded six participants who did not complete the protocol. Thus, 116 participants completed the study and were divided into two groups: the bronchiectasis (n=74) and control (n=42) groups. The physical and demographic characteristics of the two groups are shown in Table 1.

According to E-FACED, 34% of the individuals in the bronchiectasis group were classified as having mild disease, 45% as having moderate disease, and 8% as having severe disease. Fifty-one of 74 individuals were regular users of long-acting bronchodilators, 43 used macrolides, 26 used inhaled corticoids, 36 used a gastric protector, 17 used nasal corticoids, and none were on long-term prophylactic antibiotic therapy. The most prevalent comorbidities among the individuals were systemic hypertension (8%) and diabetes mellitus (7%).

The values obtained for systemic inflammatory marker, oxidant, and antioxidant levels are presented as medians and interquartile intervals. IL-6 and IL-10 levels were increased in adults with bronchiectasis. Similarly, adults with bronchiectasis presented higher carbonyl and anion superoxide levels. In contrast, catalase levels were reduced (Table 2).

Walking distance in the ISWT for both the absolute and percentage of predicted values (*p*<0.001) was reduced in adults with bronchiectasis (Table 3). There was also a significant reduction in the VO_2_ peak (*p*<0.001) and daily physical activity level (Table 3).

A statistically significant correlation was found between the aerobic capacity (absolute values) and IL-1β, IL-6, TNF-α, and TBARS (r=-0.408 and *p*=0.001; r=-0.308 and *p*=0.013; r=-0.277 and *p*=0.026; and r=0.314 and *p*=0.011, respectively). In addition, negative correlations were found between VO_2_ and IL-1β and carbonyl levels when evaluated as percentage of predicted values (r=-0.290 and r=0.379, respectively) and for workload when evaluated as percentage of predicted and absolute values (r=-0.394, r=-0.343 and r=-0.290, respectively). A weaker correlation was found between catalase activity and functional capacity and between catalase activity and the total number of steps taken (r=0.260 and r=0.295, respectively). Additionally, walking distance presented a positive correlation with TAC (r=0.235). However, no correlations were found for any other inflammatory or OS markers.

Table 4 shows a comparison between inflammatory markers and OS according to the severity of bronchiectasis.

There were no significant differences in the levels of inflammatory markers or OS markers between individuals who were on long-term macrolide treatment and those who were not on macrolide treatment (Table 5).

## DISCUSSION

The present study showed that compared to healthy controls, adults with bronchiectasis who were clinically stable presented increased levels of IL-6, IL-10, carbonylated proteins, and superoxide anion, and decreased catalase activity. Interestingly, the levels of cytokines (IL-1β, IL-6, and TNF-α) and biomarkers (TBARS and carbonyls) showed significant correlations with VO_2_. Additionally, catalase activity correlated with the functional capacity and with the total number of steps walked in adults with bronchiectasis.

Evaluation of inflammatory markers and OS in adults with bronchiectasis has been performed previously, but those studies produced inconsistent results (4,6-8,25,26). In this study, we found no differences in the plasma levels of IL-1β, IL-8 and TNF-α between the study groups. These findings are similar to those of previous studies (4,6,26). In a previous study, the serum levels of IL-8 in adults with bronchiectasis were not significantly different from those in the control group, and TNF-α was not detectable in either group (26). However, another study showed increased levels of TNF-α in adults with severe bronchiectasis when comparison to the controls. That study also demonstrated that disease severity positively correlated with systemic inflammation levels (6).

Interestingly, increased plasma levels of the immunomodulatory and anti-inflammatory cytokine IL-10 were found in adults with bronchiectasis compared to those without bronchiectasis. This finding is similar to the results reported by Bergin et al. IL-10 acts as an anti-inflammatory agent in part by inhibiting the activation of T lymphocytes, resulting in decreased production of proinflammatory cytokines (8).

In contrast, the levels of IL-1β were significantly higher in the bronchiectasis group than in the control group. However, increased levels of IL-1β were not detected, perhaps because milder cases of bronchiectasis were analyzed (forced expiratory volume in 1 second [FEV1]% predicted: 79±21). The bronchiectasis participants enrolled in the present study included more severe cases (FEV1% predicted: 53±26) (4).

It is worth noting that individuals with bronchiectasis present a high prevalence of bacterial infection; this is associated with an exaggerated neutrophilic response that perpetuates the release of inflammatory mediators that play a central role in bronchial destruction. In addition, previous studies have demonstrated that increased levels of circulating proinflammatory cytokines may indicate the presence of the most severe form of the disease (27-29). Similarly, the most severe form of bronchiectasis is followed by a more intense inflammatory process, represented, for instance, by increased levels of TNF-α in a colonized group compared with a noncolonized group (4). In this context, Martínez-García et al. observed that increased levels of TNF-α are also associated with impaired quality of life and a high prevalence of exacerbation (6).

Concerning systemic OS, we observed no increase in H_2_O_2_ in the blood of adults with bronchiectasis relative to those without bronchiectasis. This is an interesting finding because a previous study found increased levels of H_2_O_2_ in the breath condensate and bronchoalveolar lavage fluid in such patients (11,30). Altogether, these findings suggest that bronchiectasis may present increased pulmonary but not systemic OS since the pathophysiology of bronchiectasis involves OS (30). One possible explanation for these findings is that OS reactions occur rapidly at the cellular level, making them difficult to detect in the plasma compared to the lungs (12).

In the present study, we observed an increased level of catalase activity in adults with bronchiectasis, possibly indicating a decreased rate of cellular degradation of this enzyme (12). On the other hand, no difference was found in the levels of TBARS or TAC, reinforcing the findings on catalase activity levels. In this context, Olveira et al. measured the levels of catalase, TAC, TBARS, glutathione peroxidase, and antioxidant vitamins in a group of individuals with bronchiectasis (FEV_1_% predicted: 73.6±21.2) (12). The authors found higher levels of catalase, TAC, and TBARS in the bronchiectasis group than in the control group. Although they found significantly different values for TAC and TBARS, only adults with bronchiectasis with mild pulmonary impairment were analyzed (FEV_1_% predicted: 73.6 *vs.* 52.5 in the present study). The observed differences in TAC and TBARS differed from those found in our study, and this can also be explained by the differences in the techniques employed. For example, in the study by Olveira et al., the biomarkers were measured using a commercial kit, which is a more sensitive method (12).

Although not expected, the other inflammatory biomarkers evaluated in this study did not differ between adults with bronchiectasis and the controls. This finding may reflect a difference in systemic and nonlocal markers since studies have shown that plasma levels may not be detectable (20). In addition, bronchiectasis has a complex pathophysiology that leads to normal, restrictive, or obstructive pulmonary function (31). In addition, from the perspective of gas exchange, bronchiectasis can cause intermittent or nonintermittent hypoxemia during exertion, making it difficult to analyze the impact of severity on inflammatory mediators and OS.

In this study, no differences in the levels of inflammatory markers or OS were found among patients with bronchiectasis of different degrees of severity (Table 4). This finding can be explained by the relatively mild severity of the disease within our sample of individuals with bronchiectasis. However, a previous study showed that bronchiectasis severity scores exhibit a correlation with C-reactive protein levels (FEV_1_% predicted: 55.5±21.2) (32).

Previous studies correlated inflammatory mediators with lung function; however, no study has correlated the inflammatory status with exercise capacity. In a study by Wilson et al. (5), correlations between inflammation (total counts of leukocytes and neutrophils and immunoglobulin A, G, and M levels) and pulmonary function ranged from -0.27 to -0.35. Although the markers analyzed in that study were not similar to the markers analyzed in our study, the correlation was negative, showing that a higher inflammatory level was correlated with a worse lung function (4). Previous studies have also demonstrated associations of TNF-α and IL-8 levels with computed tomography severity scores (6) in adults with bronchiectasis.

Our study investigated the correlation among inflammatory markers (IL-1β and IL-6), aerobic capacity, and the number of steps taken and demonstrated that the higher an individual’s inflammatory level, the worse is his or her aerobic capacity. This result should be appreciated since VO_2_ is a variable that reflects the integrity of the respiratory, cardiovascular, and musculoskeletal systems and their integration. Similar to the results obtained in our sample, Harris et al. observed that increased levels of IL-6 in the elderly population may be related to a reduced functional capacity and reduced survival (33). IL-6 also plays an important biological role both in the acute phase of inflammation and its progression to the chronic phase. A previous study reported that higher levels of IL-6 were correlated with more frequent the exacerbations in individuals with COPD (34).

Studies investigating OS in bronchiectasis are scarce, especially those relating it to exercise capacity. Our study demonstrates an association among VO_2_ and TBARS, carbonyl, and nitrite levels. In addition, antioxidant levels (TAC and catalase activity) correlated with functional capacity. An increase in the production of free radicals can lead to an imbalance between oxidant action and antioxidants, which depends on the functional capacity and intensity of exercise. Physical exercise may increase the level of production of ROS due to alterations in mitochondrial chain reactions and probable ischemia of peripheral muscles (35).

Some limitations of our study should be considered. Individuals with bronchiectasis were recruited from a single center, although they were from different regions of the country. Most participants had mild severity of bronchiectasis; however, they presented with lung function (FEV_1_% predicted: 52.5±25.7) that was worse than the participants in Angrill et al. (4), Wilson et al. (5), and Martínez-García et al. studies (FEV_1_% predicted: 79±21, 63.0±27.0% and 62.8±19.9, respectively) (6). The inflammatory and oxidative states of the participants were not measured using bronchoalveolar lavage samples. However, a previous study has shown a relationship between bronchoalveolar lavage and systemic measurements of inflammatory, and oxidative states (4).

In conclusion, adults with bronchiectasis have high levels of proinflammatory cytokines; however, OS biomarkers in this population and the control group were similar. In addition, inflammatory cytokines and OS are associated with aerobic capacity and the number of steps walked.

## AUTHOR CONTRIBUTIONS

De Camargo AA contributed to conceptualization, data curation, funding acquisition, and manuscript writing and original drafting. De Castro RAS contributed to the study conception, data curation and design. Vieira RP contributed to methodology, resources, and manuscript writing and original drafting.

Oliveira-Júnior MC contributed to methodology, resources and funding. Araujo AA, De Angelis K, Rached SZ, Athanazio RA and Stelmach R contributed to methodology, resources, and writing of the original draft. Dal Corso S contributed to conceptualization, methodology, funding acquisition, formal analysis, and writing of the original draft.

## Figures and Tables

**Table 1:** Characteristics of the individuals with bronchiectasis and healthy controls.

Variables	Bronchiectasis group(n=74)	Control group(n=42)	Mean difference(95% CI)	*p*
Age, years	49±15	44±17	-5.2 (-11.1-0.7)	0.084
Female sex, n (%)	42 (57)	28 (67)	-	-
BMI, kg/m^2^	24.7±5.2	26.2±4.9	1.5 (-0.4-3.5)	0.121
FVC, l	2.3±0.9	3.6±1.0	1.3 (1.0-1.7)	<0.001
FVC, % of pred.	68.1±22.2	96.3±12.9	28.2 (21.7-34.6)	<0.001
FEV_1_, l	1.4±0.7	3.0±0.8	1.6 (1.3-1.9)	<0.001
FEV_1_, % of pred.	52.5±25.6	95.9±14.0	43.4 (36.1-50.7)	<0.001
FEV_1_/FVC	0.6±0.2	0.8±0.1	0.2 (0.2-0.3)	<0.001
mMRC	2 (1-3)	0 (0-0)	-	<0.001
Exacerbation, n	45	-	-	-

%: percentage. kg: kilogram. m: meter. cm: centimeter. BMI: body mass index. kg/m^2^: kilograms per square meter. FVC: forced vital capacity. l: liter. % of pred.: percentage of predicted. FEV1: forced expiratory volume in one second. mMRC: Modified Medical Research Council.

**Table 2:** Plasma markers of inflammatory cytokine levels and oxidative stress in individuals with bronchiectasis and the controls.

Mediators	Bronchiectasis group(n=74)	Control group(n=42)	*p*
Inflammatory and anti-inflammatory			
IL-1β, pg/ml	32.63 (14.79-63.72)	19.19 (16.37-28.70)	0.064
IL-6, pg/ml	120.54 (62.04-177.35)	8.76 (6.81-83.65)	<0.001
IL-8, pg/ml	167.05 (104.55-250.40)	169.22 (112.99-238.58)	0.872
TNF-α, pg/ml	198.30 (50.89-284.32)	100.55 (56.13-269.96)	0.730
IL-10, pg/ml	58.44 (26.14-100.27)	18.13 (11.49-40.28)	<0.001
Oxidants and antioxidants			
Catalase activity, nmol/mg protein	0.03 (0.02-0.05)	0.04 (0.03-0.07)	<0.001
TBARS, µmol/mg protein	0.68 (0.29-1.40)	0.34 (0.27-0.94)	0.079
Carbonyl, nmol/mg protein	1.78 (1.61-1.99)	1.58 (1.04-1.84)	0.001
Nitrite, µmol/mg protein	0.54 (0.32-0.77)	0.50 (0.26-0.92)	0.863
H_2_O_2_, µM of H_2_O_2_	43.89 (24.07-85.94)	45.96 (23.44-64.54)	0.756
O_2_-, µmol/mg protein	43.87 (35.14-65.17)	38.93 (32.98-50.84)	0.046
TAC, µM of trolox	112.07 (81.56-140.72)	102.61 (80.96-141.34)	0.838

IL: Interleukin; β: beta; pg/ml: picograms per milliliter; TNF-α: tumor necrosis factor alpha; µmol/mg: micromoles per milligram; TBARS: thiobarbituric acid reactive substance; H2O_2_: hydrogen peroxide; O_2_-: superoxide anion; TAC: total antioxidant capacity. Data are expressed as median and interquartile interval.

**Table 3:** Comparison of functional and exercise capacity between the bronchiectasis and control groups.

Variables	Bronchiectasis group(n=74)	Control group(n=42)	Mean difference(95% CI)
Functional capacity			
ISWT, m	439.5±140.8	654.4±215.7	215.0 (140.8-289.1)^*^
ISWT, % pred.	55.1±17.8	75.7±14.8	20.6 (14.1-27.0)^*^
Aerobic capacity			
VO_2_ peak, ml kg^-1^ min^-1^	17.1±5.5^a^	23.2±7.3	6.1 (3.7-8.6)^*^
VO_2_ peak, % pred.	62.8±16.5^a^	83.6±19.5	20.7 (13.8-27.7)^*^
VE, L/min	39.2±14.2^a^	58.9±21.9	19.7 (12.1- 27.3)^*^
VE, % pred.	37.1±13.0^a^	47.7±14.3	10.6 (5.3-15.9)^*^
Work, W	72.7±34.1	125.4±51.6	52.7 (34.9-70.5)^*^
Work, % pred.	63.8±22.3	95.2±23.4	31.4 (13.5-28.0)^*^
HR, bpm	140±18	159±24	18.6 (10.9-26.2)^*^
HR, % pred.	82.4±8.8	90.8±12.5	8.4 (4.5-12.4)^*^
Pulse of oxygen, ml/beat	12.0±3.0^a^	14.4±3.5	2.5 (1.2-3.7)^*^
Physical activity in daily life			
No. of steps/day	9,392.1±4095.0	11,016.4±3979.9	2,624.3 (1,072.8-4,175.8)^#^

a: n=9 adults with bronchiectasis were dependent on oxygen; ISWT: incremental shuttle walking test; m: meters; % pred.: percentage of predicted; VO_2_: oxygen consumption; ml.kg^-1^.min^-1^: milliliters per kilogram per minute; VE: ventilation; L/min: liters per minute; W: watts; HR: heart rate; bpm: beats per minute; ml/beat: milliliters per beat; No. of steps/day: total number of steps. *: *p*<0.001; #: *p*=0.001.

**Table 4:** Comparison of plasma markers of inflammatory cytokine levels and oxidative stress in subjects with bronchiectasis according to severity of disease (E-FACED).

Mediators	Bronchiectasis, mild(n=42)	Bronchiectasis,moderate and severe(n=27)	*p*
Inflammatory and anti-inflammatory			
IL-1β, pg/ml	32.19 (14.79-64.20)	28.35 (13.51-68.84)	0.293
IL-6, pg/ml	128.70 (53.29-163.06	127 (65.11- 266.36)	0.384
IL-8, pg/ml	165.73 (97.86-263.44)	192.20 (117.75-321.66)	0.302
TNF-α, pg/ml	197.95 (47.45-305.87)	204.50 (50.02-269.21)	0.210
IL-10, pg/ml	56.62 (26.09-95.12)	65.24 (30.23-114.96)	0.824
Oxidations and antioxidants			
Catalase activity, nmol/mg protein	0.03 (0.02-0.05)	0.03 (0.02-0.05)	0.215
TBARS, µmol/mg protein	0.68 (0.28-1.34)	0.68 (0.33-1.53)	0.349
Carbonyl, nmol/mg protein	1.79 (1.60-1.98)	1.73 (1.61-2.46)	0.426
Nitrite, µmol/mg protein	0.55 (0.37-0.93)	0.60 (0.27-0.75)	0.438
H_2_O_2_, µM of H_2_O_2_	44.81 (31.29-86.67)	41.85 (25.56-87.06)	0.793
O_2_-, µmol/mg protein	44.08 (34.04-61.64)	43.65 (34.97-68.68)	0.415
TAC, µM of trolox	112.07 (79.60-138.88)	133.83 (83.52-143.66)	0.861

IL: Interleukin; β: beta; pg/ml: picograms per milliliter; TNF-α: tumor necrosis factor alpha; µmol/mg: micromoles per milligram; TBARS: thiobarbituric acid reactive substance; H2O_2_: hydrogen peroxide; O_2_-: superoxide anion; TAC: total antioxidant capacity. Data are expressed as median and interquartile interval.

**Table 5:** Plasma markers of inflammatory cytokine levels and oxidative stress in individuals with bronchiectasis treated with or without macrolides.

Mediators	Macrolide treatment(n=46)	No macrolide treatment(n=28)	*p*
Inflammatory and anti-inflammatory			
IL-1β, pg/ml	27.17 (13.25-53.65)	33.56 (16.03-67.64)	0.29
IL-6, pg/ml	93.19 (51.99-143.87)	137.18 (60.14-189.88)	0.38
IL-8, pg/ml	182.83 (118.44-257.47)	162.56 (97.86-236.22)	0.30
TNF-α, pg/ml	157.60 (57.29-267.01)	222.55 (65.40-322.30)	0.21
IL-10, pg/ml	58.87 (22.48-121.51)	57.12 (26.13-89.36)	0.82
Oxidation and antioxidants			
Catalase activity, nmol/mg protein	0.03 (0.02-0.05)	0.02 (0.02-0.04)	0.22
TBARS, µmol/mg protein	0.53 (0.28-1.23)	0.82 (-0.28-1.63)	0.35
Carbonyl, nmol/mg protein	1.78 (1.63-2.27)	1.79 (1.60-1.92)	0.43
Nitrite, µmol/mg protein	0.52 (0.32-0.78)	0.64 (0.31-0.85)	0.44
H_2_O_2_, µM of H_2_O_2_	41.85 (26.77-68.52)	44.07 (16.85-97.78)	0.79
O_2_-, µmol/mg protein	46.36 (37.02-72.98)	40.74 (35.07-64.83)	0.42
TAC, µM of trolox	107.70 (85.23-137.04)	112.06 (68.18-141.66)	0.86

L: Interleukin; β: beta; pg/ml: picograms per milliliter; TNF-α: tumor necrosis factor alpha; µmol/mg: micromoles per milligram; TBARS: thiobarbituric acid reactive substance; H2O_2_: hydrogen peroxide; O_2_-: superoxide anion; TAC: total antioxidant capacity. Data are expressed as median and interquartile interval.

## APPENDIX

### Oxidative Stress Profile

#### Proteins

Proteins were quantified by the method of Lowry et al., which uses serial dilutions of bovine albumin solution [1 mg/mL] as a standard; 10 μL of sample was used. Such quantifications were used to calculate concentrations of carbonyl, catalase activity, TBARS, superoxide anion, TRAP, H2O2 and Nitrite.

#### Oxidatively Modified Proteins

The protein damage was determined by protein carbonyls measurements, using 200 *μ*L of sample. Plasma samples were incubated with 2,4-dinitrophenylhydrazine (DNPH 10 mM) in a 2.5 M HCl solution for 1h at room temperature in the dark. Samples were vortexed every 15 min. Subsequently, a 20% trichloroacetic acid (w/v) solution was added and the solution was incubated on ice for 10 min and centrifuged for 5 min at 1000 g to collect protein precipitates. An additional wash was performed with 10% trichloroacetic acid (w/v). The pellet was washed three times with ethanol/ethyl acetate (1:1) (v/v). The final precipitates were dissolved in 6 M guanidine hydrochloride solution and incubated for 10 min at 37°C, and the absorbance was measured at 360 nm.

#### Catalase activity

Catalase activity (CAT) was determined by measuring the decrease in H_2_O_2_ absorbance at 240 nm.

#### Thiobarbituric Acid Reactive Substances (TBARS)

Plasma lipid peroxide levels were determined by measuring TBARS, a common method for measuring the concentration of malondialdehyde, a breakdown product of oxidized lipids. For the TBARS assay, using 250 μL of sample, trichloroacetic acid (10%, w/v) was added to the homogenate to precipitate proteins and to acidify the samples. This mixture was then centrifuged (4000 rpm, 10 min), the protein-free sample was extracted, and thiobarbituric acid (0.67%, w/v) was added to the reaction medium. The tubes were placed in a water bath (100°C) for 30 min. Absorbance’s were measured at 535 nm using a spectrophotometer.

#### Superoxide anion

The superoxide anion was determined in LV homogenates by calculating the rate of oxidation of adrenaline at 480nm, as described by McCord and Fridovich.

#### Total radical-trapping potential (TRAP)

The TRAP represents the total antioxidant capacity, which was measured by chemical reaction: 150µl of the plasma, 150µl of the sodium dodecyl sulfate 8.1% (P/V), 300µl of Trichloroacetic Acid (Vetec Quimica Fina Ltda.) 20% (P/V) and 500µl of Thiobarbituric Acid (Sigma-Aldrich Corporation). The mixture was incubated for 20-30 minutes at 95°C, forming a pink compound and then cooled on ice, followed by centrifugation at 4000rpm for 5 min (Eppendorf AG, Germany). The supernatant (200µl) was transferred to an ELISA plate. The reading was made at 535nm wavelength in an ELISA Plate reader (Robonik, India).

#### Hydrogen peroxide (H_2_O_2_)

Hydrogen peroxide (H_2_O_2_) was measured through oxidation of phenol mediated by red radish peroxidase, resulting in the formation of a compound measurable at 630nm. It was performed a curve with distilled H_2_O, H_2_O_2_ at 250µM, Radish peroxidase solution (RPS) composed of dextrose buffer, phenol red (Sigma-Aldrich Corporation) and Radish Peroxidase of type II (Sigma-Aldrich Corporation), and sodium hydroxide (NaOH). Plasma (70µl) and 180µl of RPS were added in the ELISA plate and incubated for 25 min at room temperature. After this period 5µl of NaOH was added and plate was read in a ELISA plate reader (Robonik, India).

#### Nitrite Concentrations

The nitrite levels were measured by the reaction of the samples with the Griess reagent microplates (96 wells) in the ELISA reader device. Total nitrite levels were analyzed in tissue samples from the reaction with 50ml of Griess reagent. Nitrite was calculated from a standard curve of absorbance in 592nm.

## References

[1] King PT (2009). The pathophysiology of bronchiectasis. Int J Chron Obstruct Pulmon Dis.

[2] Saleh AD, Chalmers JD, De Soyza A, Fardon FC, Koustas SO, Scott J (2017). The heterogeneity of systemic inflammation in bronchiectasis. Respir Med.

[3] Fuschillo S, De Felice A, Balzano G (2008). Mucosal inflammation in idiopathic bronchiectasis: cellular and molecular mechanisms. Eur Respir J.

[4] Angrill J, Agustí C, De Celis R, Filella X, Raãó A, Elena M (2001). Bronchial inflammation and colonization in patients with clinically stable bronchiectasis. Am J Respir Crit Care Med.

[5] Wilson CB, Jones PW, O'Leary CJ, Hansell DM, Dowling RB, Cole PJ (1998). Systemic markers of inflammation in stable bronchiectasis. Eur Respir J.

[6] Martínez-García MA, Perpiãá-Tordera M, Román-Sánchez P, Soler-Cataluãa JJ, Carratalá A, Yago M (2008). [The association between bronchiectasis, systemic inflammation, and tumor necrosis factor alfa]. Arch Bronconeumol.

[7] Gaga M, Bentley AM, Humbert M, Barkans J, O’Brien F, Wathen CG (1998). Increases in CD4+ T lymphocytes, macrophages, neutrophils and interleukin 8 positive cells in the airways of patients with bronchiectasis. Thorax.

[8] Bergin DA, Hurley K, Mehta A, Cox S, Ryan D, O’Neill SJ (2013). Airway inflammatory markers in individuals with cystic fibrosis and non-cystic fibrosis bronchiectasis. J Inflamm Res.

[9] Cantin AM, White TB, Cross CE, Forman HJ, Sokol RJ, Borowitz D (2007). Antioxidants in cystic fibrosis. Conclusions from the CF antioxidant workshop, Bethesda, Maryland, November 11-12, 2003. Free Radic Biol Med.

[10] Gea J, Agustí A, Roca J (2013). Pathophysiology of muscle dysfunction in COPD. J Appl Physiol (1985).

[11] Loukides S, Horvath I, Wodehouse T, Cole PJ, Barnes PJ (1998). Elevated levels of expired breath hydrogen peroxide in bronchiectasis. Am J Respir Crit Care Med.

[12] Olveira G, Olveira C, Dorado A, García-Fuentes E, Rubio E, Tinahones F (2013). Cellular and plasma oxidative stress biomarkers are raised in adults with bronchiectasis. Clin Nutr.

[13] José A, Ramos TM, de Castro RAS, de Oliveira CS, de Camargo AA, Athanazio RA (2018). Reduced Physical Activity With Bronchiectasis. Respir Care.

[14] de Camargo AA, Boldorini JC, Holland AE, de Castro RAS, Lanza FC, Athanazio RA (2018). Determinants of Peripheral Muscle Strength and Activity in Daily Life in People With Bronchiectasis. Phys Ther.

[15] Allaire J, Maltais F, Doyon JF, Noel M, LeBlanc P, Carrier G (2004). Peripheral muscle endurance and the oxidative profile of the quadriceps in patients with COPD. Thorax.

[16] Terpstra LC, Biesenbeek S, Altenburg J, Boersma WG (2019). Aetiology and disease severity are among the determinants of quality of life in bronchiectasis. Clin Respir J.

[17] Li L, Zhang J, Li Z, Zhang C, Bi J, Zhou J (2021). Airway microbiota is associated with the severity of non-CF bronchiectasis. Clin Respir J.

[18] Hill AT, Haworth CS, Aliberti S, Barker A, Blasi F, Boersma W (2017). Pulmonary exacerbation in adults with bronchiectasis: a consensus definition for clinical research. Eur Respir J.

[19] Sociedade Brasileira de Pneumologia e Tisiologia (2002). Diretrizes para testes de função pulmonar. J Pneumol.

[20] Singh SJ, Morgan MD, Scott S, Walters D, Hardman AE (1992). Development of a shuttle walking test of disability in patients with chronic airways obstruction. Thorax.

[21] Neder JA, Nery LE, Castelo A, Andreoni S, Lerario MC, Sachs A (1999). Prediction of metabolic and cardiopulmonary responses to maximum cycle ergometry: a randomised study. Eur Respir J.

[22] Bradley JM, Wilson JJ, Hayes K, Kent L, McDonough S, Tully MA (2015). Sedentary behaviour and physical activity in bronchiectasis: a cross-sectional study. BMC Pulm Med.

[23] Mahler DA, Wells CK (1988). Evaluation of clinical methods for rating dyspnea. Chest.

[24] Martinez-Garcia MA, Athanazio RA, Girón R, Máiz-Carro L, de la Rosa D, Olveira C (2017). Predicting high risk of exacerbations in bronchiectasis: the E-FACED score. Int J Chron Obstruct Pulmon Dis.

[25] Ayhan G, Tas D, Yilmaz I, Okutan O, Demirer E, Ayten O (2014). Relation between inflammatory cytokine levels in serum and bronchoalveolar lavage fluid and gene polymorphism in young adult patients with bronchiectasis. J Thorac Dis.

[26] Ergan Arsava B, Cöplü L (2011). Does airway colonization cause systemic inflammation in bronchiectasis?. Tuberk Toraks.

[27] Puren AJ, Feldman C, Savage N, Becker PJ, Smith C (1995). Patterns of cytokine expression in community-acquired pneumonia. Chest.

[28] Igonin AA, Armstrong VW, Shipkova M, Lazareva NB, Kukes VG, Oellerich M (2004). Circulating cytokines as markers of systemic inflammatory response in severe community-acquired pneumonia. Clin Biochem.

[29] Norman D, Elborn JS, Cordon SM, Rayner RJ, Wiseman MS, Hiller EJ (1991). Plasma tumour necrosis factor alpha in cystic fibrosis. Thorax.

[30] Loukides S, Bouros D, Papatheodorou G, Lachanis S, Panagou P, Siafakas NM (2002). Exhaled H(2)O(2) in steady-state bronchiectasis: relationship with cellular composition in induced sputum, spirometry, and extent and severity of disease. Chest.

[31] Habesoglu MA, Ugurlu AO, Eyuboglu FO (2011). Clinical, radiologic, and functional evaluation of 304 patients with bronchiectasis. Ann Thorac Med.

[32] Coban H, Gungen AC (2017). Is There a Correlation between New Scoring Systems and Systemic Inflammation in Stable Bronchiectasis?. Can Respir J.

[33] Harris TB, Ferrucci L, Tracy RP, Corti MC, Wacholder S, Ettinger WH  (1999). Associations of elevated interleukin-6 and C-reactive protein levels with mortality in the elderly. Am J Med.

[34] Wedzicha J, Seemungal T, MacCallum PK, Paul EA, Donaldson GC, Bhowmik A (2000). Acute exacerbations of chronic obstructive pulmonary disease are accompanied by elevations of plasma fibrinogen and serum IL-6 levels. Thromb Haemost.

[35] Deskur E, Przywarska L, Dylewicz P, Szczesniak L, Rychlewski T, Wilk M (1998). Exercise-induced increase in hydrogen peroxide plasma levels is diminished by endurance training after myocardial infarction. Int J Cardiol.

